# A novel pyroptosis-related lncRNA signature for prognostic prediction in patients with lung adenocarcinoma

**DOI:** 10.1080/21655979.2021.1972078

**Published:** 2021-09-07

**Authors:** Jiahang Song, Yuanyuan Sun, Hui Cao, Zhengcheng Liu, Lei Xi, Changqing Dong, Rusong Yang, Ye Shi

**Affiliations:** a Department of Thoracic Surgery, Nanjing Chest Hospital, Nanjing, Jiangsu, China; b Department of Radiation Oncology, The First Affiliated Hospital of Nanjing Medical University, Nanjing, Jiangsu, China; c Department of Thoracic Surgery, The Affiliated Nanjing Brain Hospital of Nanjing Medical University, Nanjing, Jiangsu, China; d Department of Thoracic Surgery, The Pulmonary Nodule Diagnosis and Treatment Research Center of Nanjing Medical University, Nanjing, Jiangsu, China

**Keywords:** Lung adenocarcinoma, pyroptosis, lncRNA, bioinformatics, prognostic signature, TCGA

## Abstract

Lung adenocarcinoma (LUAD) has been the major cause of tumor-associated mortality in recent years and has a poor prognosis. Pyroptosis is regulated via the activation of inflammasomes and participates in tumorigenesis. However, the effects of pyroptosis-related lncRNAs (PRlncRNAs) on LUAD have not yet been completely elucidated. Therefore, we attempted to systematically explore patterns of cell pyroptosis to establish a novel signature for predicting LUAD survival. Based on TCGA database, we set up a prognostic model by incorporating PRlncRNAs with differential expression using Cox regression and LASSO regression. Kaplan–Meier analysis was conducted to compare the survival of LUAD patients. We further simplified the risk model and created a nomogram to enhance the prediction of LUAD prognosis. Altogether, 84 PRlncRNAs with differential expression were discovered. Subsequently, a new risk model was constructed based on five PRlncRNAs, GSEC, FAM83A-AS1, AL606489.1, AL034397.3 and AC010980.2. The proposed signature exhibited good performance in prognostic prediction and was related to immunocyte infiltration. The nomogram exactly forecasted the overall survival of patients and had excellent clinical utility. In the present study, the five-lncRNA prognostic risk signature and nomogram are trustworthy and effective indicators for predicting the prognosis of LUAD.

## Graphical abstract

**Figure:**
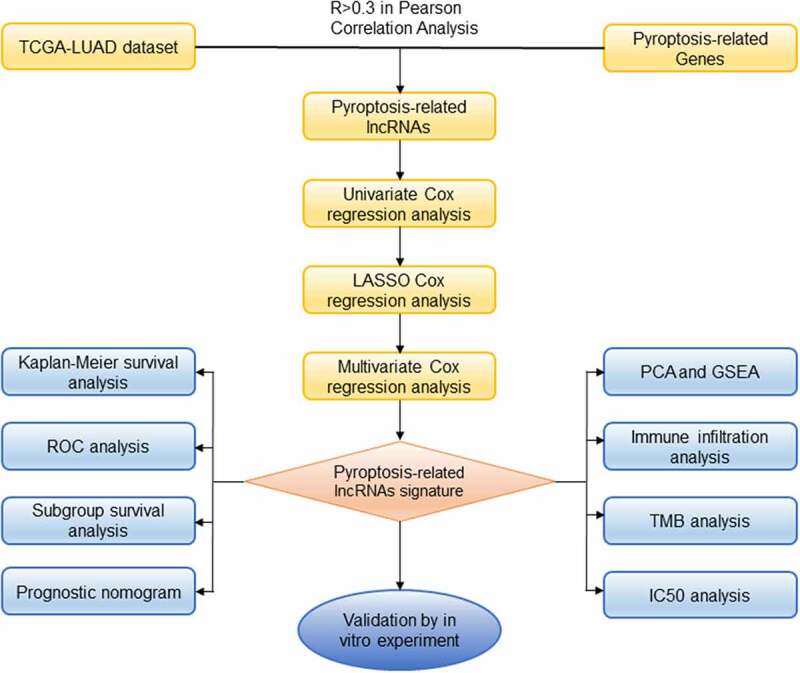

## Introduction

Lung cancer represents a fatal malignancy and a primary cause of cancer-associated mortality, with 2,206,771 new lung cancer cases and 1,796,144 deaths occurring in 2020 worldwide [1]. The most frequently observed histological subtype of lung cancer is non-small cell lung cancer (NSCLC), which is primarily classified into squamous cell carcinoma (LUSC) and adenocarcinoma (LUAD) [2]. Despite new developments in cancer therapeutic treatments for LUAD in recent years, including surgical resection, immunotherapy, chemotherapy and radiotherapy, the prognosis of LUAD continues to be frustrating, and its 5-year survival is less than 20% [3]. Therefore, there is an urgent need to identify robust biomarkers for predicting the prognosis of LUAD patients.

Long noncoding RNAs (lncRNAs) comprise at least 200 nucleotides [4]. The close interaction between lncRNAs and other cellular molecules, including DNA, mRNA, and microRNA, has been reported to be involved in multiple diseases, including cardiovascular diseases, cancers, and metabolic diseases [5–8], and has attracted increasing attention. For instance, aging-regulated lncRNAs were found to act as antiapoptotic agents in cardiomyocytes [9], and more recently, Lin et al. discovered that several lncRNAs are involved in papillary thyroid carcinoma by sponging microRNAs and modulating the PI3K/Akt and Wnt signaling pathways [10]. Moreover, accumulating evidence indicates the prognostic value of aberrantly expressed lncRNAs in multiple biological and pathological processes. However, the pyroptosis-related lncRNAs associated with cancer diagnosis and prognosis remain poorly understood, making it very urgent to identify novel biomarkers based on pyroptosis-related lncRNA expression profiles.

Pyroptosis, a newly observed proinflammatory form of programmed cell death (PCD), is distinct from apoptosis, autophagy and ferroptotic cell death [11]. The pyroptotic process is hallmarked by the rapid rupture of the plasma membrane and the release of proinflammatory cytokines. Pyroptotic cells first generate numerous vesicles under an electron microscope. After the development of these vesicles, pores are formed in the cell membrane through which the intracellular contents flow out [12]. The typical pyroptotic pathway is sparked by the activation of nucleotide-binding domain leucine-rich repeats family protein 3 (NLRP3) inflammasomes as well as the associated inflammatory response [13,14]. Several reports have verified that pyroptosis plays a critical role in tumorigenesis and cancer treatment [15–19]. Notably, various cancers, including colon cancer [20,21], gastric cancer [22], hepatocellular carcinoma [23,24], breast cancer [25,26] and lung cancer [27,28], are sensitive to pyroptosis. Pyroptosis-related genes (PRGs), such as NLRP3 [29,30], Caspase 1 (CASP1) [31], Gasdermin D (GSDMD) [11,27] and Gasdermin E (GSDME) [32], are strongly implicated in oncogenesis and tumor progression. For example, GSDMD represses the proliferation of lung cancer cells by inhibiting EGFR/Akt signaling and inducing the intrinsic mitochondrial apoptotic pathway [27]. In colon cancer, Dupaul-Chicoine et al. revealed that knocking out NLRP3 and CASP1 favored the development of cancer in a transgenic mouse model compared to wild type mice [33]. Numerous cellular molecules, such as lncRNAs, are also involved in the regulation of pyroptosis. Liu et al. suggested that lncRNA-XIST knockdown abolished the development of NSCLC by promoting pyroptotic cell death [34]. LncRNA ADAMTS9-AS2 restrained gastric cancer advancement through the pyroptosis pathway [35]. However, the effect of pyroptosis-associated lncRNAs in LUAD has not been completely clarified. Consequently, we attempted to identify a pyroptosis-related lncRNA (PRlncRNA) signature and to investigate its clinical correlation with LUAD.

While several previous reports have investigated the relationship between pyroptosis-associated lncRNAs and prognosis in patients with lung cancer, there has yet to be much systematic analysis with respect to the context of pyroptosis in LUAD, and the underlying mechanism of LUAD remains poorly understood. Here, we identified pyroptosis-associated lncRNAs and generated a novel risk signature for the prognostic prediction of LUAD, opening up new perspectives for promoting individualized treatment for LUAD patients.

## Materials and methods

### Data collection

We obtained both clinical and FPKM RNA-seq data from LUAD cases using TCGA database (https://tcga-data.nci.nih.gov/tcga/), including 535 cancer samples and 59 noncancerous samples. Next, based on patient ID, we compared the clinical data of patients to their transcriptome data. Patients who had unmatched IDs were excluded from this work. Our inclusion criteria for patients were as follows [1]: histologically diagnosed with LUAD [2]; available expression profiles; and [3] OS time greater than 30 days. Consequently, we extracted 504 patients who had sufficient gene expression profiling along with overall survival (OS) data from the TCGA dataset for subsequent analysis. Next, a total of 14 pyroptosis-related genes (CASP1, CASP3, CASP4, CASP5, PYCARD, IL18, IL1B, NLRP3, NLRC4, GSDMA, GSDMB, GSDMC, GSDMD and GSDME) were retrieved from previous research and the literature [36–38].

### Identification of differentially expressed PRlncRNAs

To identify PRlncRNAs, we used Pearson correlation to assess the associations between lncRNAs and PRGs. Typically, we selected PRlncRNAs using the thresholds of p < 0.001 and correlation coefficient |R^2^| > 0.3. Based on the Bioconductor limma package in R software [39], we compared LUAD samples and non-carcinoma samples and selected the differentially expressed lncRNAs (DElncRNAs). DElncRNAs were determined based on |log2 (fold change, FC) | >1 and false discovery rate (FDR) <0.05 thresholds. Next, differentially expressed PRlncRNAs were extracted from the DElncRNAs.

### Construction of the PRlncRNAs prognostic model

To develop an optimal PRlncRNAs prognostic model, we randomly and evenly separated patients from the entire set (n = 504) into training or internal test sets at a 1:1 ratio. First, potential prognostic lncRNAs were identified by univariate Cox regression from the training set using the threshold of p < 0.05. Subsequently, overfitting genes were reduced by least absolute shrinkage and selection operator (LASSO) regression. Finally, we established a prognostic model by multivariate Cox regression prognostic outcomes of LUAD. The risk score for LUAD cases was calculated as follows: risk score = (PRlncRNA 1 expression × coefficient) + (PRlncRNA 2 expression× coefficient) + … + (PRlncRNA n expression× coefficient). Meanwhile, the cases were classified into low- or high-risk groups based on the median value. In addition, the entire set and test set were used to validate our signature. Moreover, we utilized the R package ‘rms’ to construct a nomogram that integrated the risk score of the signature and clinical factors (age, clinical stage, T stage and N stage) [40]. Calibration curves were plotted to determine the discrimination power of the nomogram.

### Gene set enrichment analysis (GSEA)

GSEA was employed to detect the high-risk group correlated pathways and biological processes. The expressed gene sets of the low- or high-risk group together with hallmark gene sets collected based on Molecular Signatures Database v7.1 were analyzed using GSEA software. Gene sets conforming to NOM p < 0.05 and | NES |> 1 were deemed to be significant based on the User Guide of GSEA [41].

### Infiltrating immune cell analysis of the prognostic signature

We evaluated the association between the risk model and immunocyte infiltration according to the Tumor Immune Estimation Resource (TIMER, https://cistrome.shinyapps.io/timer/), which can be utilized to detect the infiltration fraction of six immune cells, including B cells, CD4 + T cells, CD8 + T cells, neutrophils, macrophages, and dendritic cells, in the tumor microenvironment [42]. Spearman’s test was used to analyze the correlations of the risk score with the infiltrating immunocytes. The significance level was set at P < 0.05.

### Tumor mutation burden analysis

LUAD mutation data (TCGA.LUAD.varscan.acb6852e-dd48-4ca5-80f2-3d1a2c7d7ceb.DR-10.0.somatic) were also acquired from TCGA database. Somatic mutations in LUAD were evaluated based on the Mutation Annotation Format (MAF) and assessed using MAF tools [43]. The tumor mutation burden (TMB) score for each LUAD case was generated using the following formula $TMB\;=\frac{total\;mutation}{total\;covered\;bases}\times 10^{6}$ [44].

### Analysis of the risk model performance in clinical chemotherapy

To assess the signature in the clinical utility of LUAD treatment, we analyzed the half inhibitory centration (IC50) of typical chemotherapy drugs in the TCGA dataset using pRRophetic R software [45]. AJCC guidelines recommend antineoplastic agents, such as cisplatin, docetaxel, doxorubicin, gemcitabine and paclitaxel, for the treatment of hepatocellular carcinoma. Thereafter, we utilized the Wilcoxon signed-rank test to detect the heterogeneous IC50 in the low-risk group relative to the high-risk group.

### Cell culture and transfection

We obtained a human lung epithelial cell line (BEAS-2B) together with human LUAD cell lines (A549 and NCI-H460) from Shanghai Institute of Biochemistry and Cell Biology, the Chinese Academy of Sciences. Then, we cultivated cells in RPMI-1640 medium that contained 10% fetal bovine serum (FBS, Gibco Company) and 10% penicillin-streptomycin (Sigma‐Aldrich), followed by incubation at 37°C and 5% CO_2_. siRNA negative control (si‐NC) and si‐GSEC were chemically synthesized by RiboBio (Guangzhou, China). We transfected si‐GSEC and its negative control (si-NC) into lung adenocarcinoma cell lines. The sense sequence of si-GSEC was 5ʹ-GGUCACAACAGUACAAAGA-3ʹ. Subsequently, Lipofectamine 3000 (Invitrogen) was utilized to transfect cells with siRNAs in line with specific protocols. Forty-eight hours post-transfection, we collected cells for subsequent experiments.

### RNA extraction and quantitative real-time PCR (qRT-PCR)

Total cellular RNA was isolated using TRIzol (Vazyme Biotech, Nanjing, China). Then, the extracted RNA was measured for concentration and purity using a BioSpec-nano spectrophotometer (Shimadzu, Japan) and was reverse transcribed to synthesize complementary DNA (cDNA) utilizing the Prime Script RT Master Mix reagent (Takara Bio, Dalian, China). Subsequently, we performed qRT-PCR using the StepOnePlus real-time PCR system (Thermo Fisher Science) and polymerase chain reaction system TB Green®PreMix Ex Taq™ (Takara Bio, Dalian, China), and the 2^-ΔΔCT^ method was used to calculate the relevant gene expression. The expression of GSEC was detected using 5ʹ-GAGTTCATTTGCTCTCTCTGGCAC-3ʹ (forward) and 5ʹ-AAGAGGAGGCCTGATGGGGATA-3ʹ (reverse) primers. GAPDH was used as a reference gene.

### Western blot analysis

Western blot analysis was conducted to determine NLRP3, cleaved caspase‑1 and GAPDH levels. Antibodies against NLRP3 (#15,101, 1:1,000), cleaved caspase‑1 (#4199, 1:1,000) and GAPDH (#5174, 1:1,000) were provided by Cell Signaling Technology (CST, Danvers, MA, US). Each protein’s expression was detected using Super ECL Plus Detection Reagent (Millipore) on a Bio-Imaging System (Bio-Rad, USA).

### Cell counting kit-8 (CCK-8) assay

We measured cell proliferation using a CCK-8 assay (Beyotime, Shanghai, China) following specific protocols. Thereafter, cells (2000/well) were inoculated into 96-well plates and cultured in RPMI-1640 medium containing 10% FBS. At a fixed time of day, we added CCK-8 solution to each well and incubated the cells at 37°C for an additional 2 h. The absorbance values were measured at 450 nm using a microplate spectrophotometer (Thermo, USA) and were used to determine the capability of LUAD cell proliferation.

### Colony formation assay

To perform the colony formation assay, transfected cells (300/well) were plated into 6-well plates and cultured for 14 days in RPMI-1640 medium containing 10% FBS. Next, 1% formaldehyde was used to fix proliferating cell colonies, whereas 1% crystal violet was applied for staining. The number of colonies that contained at least 50 cells was calculated, and photos were taken.

## Statistical analysis

R software (3.6.3) and GraphPad (8.0) were employed for all statistical data analyses. Differences in OS between high-risk patients and low-risk patients were assessed using Kaplan–Meier analysis as well as log-rank tests. Additionally, independent predictors were identified by univariate and multivariate Cox regression. Moreover, we plotted time-dependent receiver operating characteristic (t-ROC) curves to evaluate the predictive performance of our established prognostic signature. P < 0.05 was set as the significance level.

## Results

In the present study, our proposed novel pyroptosis-related lncRNAs signature was composed of five lncRNAs: GSEC, FAM83A-AS1, AL606489.1, AL034397.3 and AC010980.2. The pyroptosis-related risk model could ameliorate the prediction of LUAD prognosis. Cox relative regression methods indicated the independence of risk score generated from the risk model. Moreover, we simplified the constructed signature to set up a nomogram by combining risk score and other clinical traits. Immune microenvironment analysis, GSEA, and chemotherapy drugs analysis were utilized to exploit the clinical potency of the risk signature. Finally, we selected the lncRNA GSEC to valid our signature by in vitro experiments, including qRT-PCR, CCK-8 assay, colony formation assay and western blot analysis.

### Identification of differentially expressed PRlncRNAs

**Figure 1.**
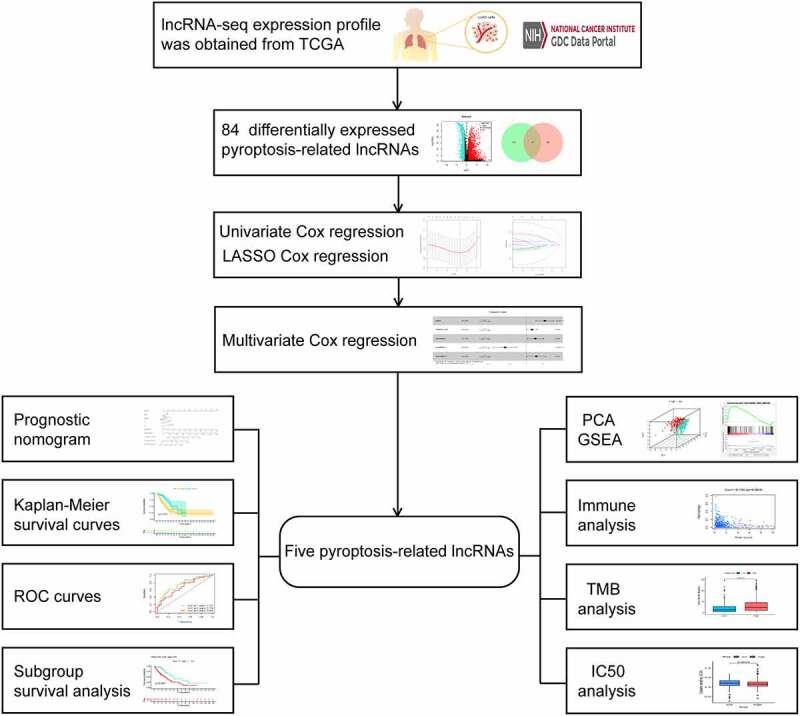
Infographic flowchart of the whole study

**Figure 2.**
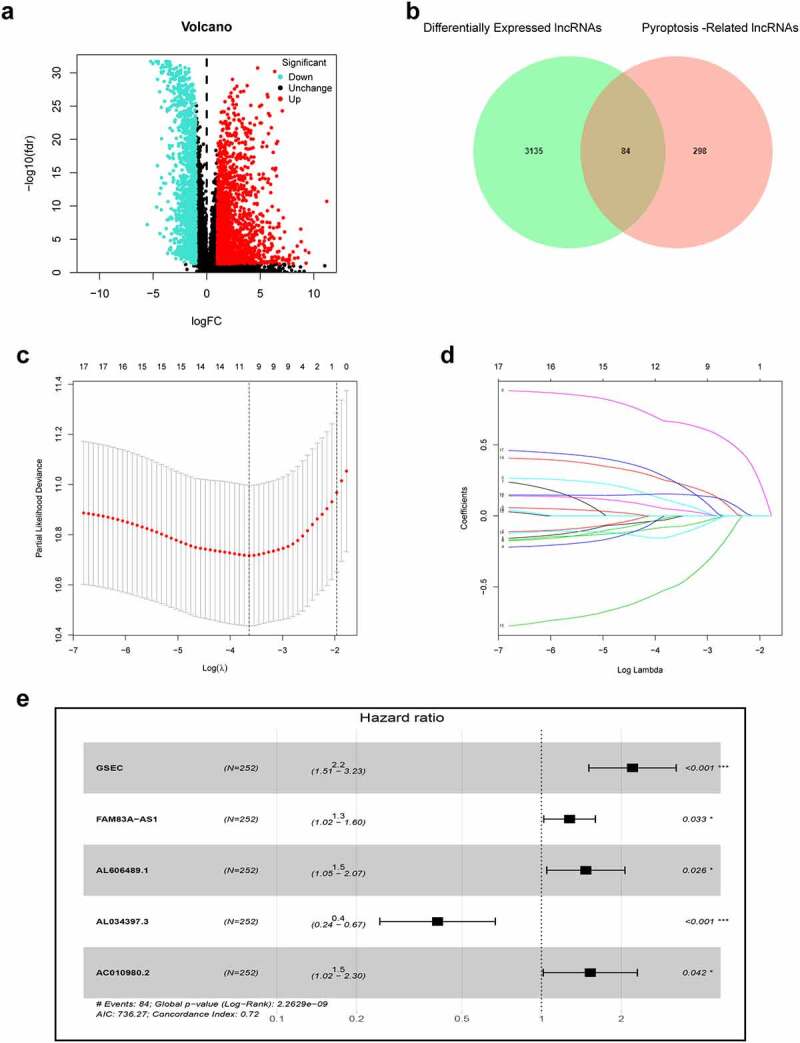
Construction of prognostic pyroptosis-related risk model composed of five lncRNAs. (a) Volcano plot presenting differentially expressed lncRNAs (DElncRNAs) discovered from LUAD tissues compared with non-carcinoma samples from TCGA dataset; (b) The Venn diagram of genes among DElncRNAs list and pyroptosis-related lncRNAs; (c-d) Lasso Cox regression analysis showing that 11 out of the 84 pyroptosis-related lncRNAs were good candidates for constructing the prognostic signature; (e) Forest plot presenting the HRs for the pyroptosis-associated prognosis model containing five lncRNAs

The infographic flowchart of the whole study is shown in Figure 1. Based on the 14 pyroptosis-related genes (PRGs), we identified 382 PRlncRNAs by Pearson correlation analysis of lncRNA levels and PRG levels in the LUAD samples (Pearson correlation coefficient > 0.3, p < 0.001). Using cutoff values of |log_2_FC| > 1 and P < 0.05, 3219 DElncRNAs were identified between 535 LUAD and 59 noncancerous samples (Figure 2a). By overlapping the PRlncRNAs and DElncRNAs of LUAD, we identified 84 significantly differentially expressed pyroptosis-related lncRNAs (DEPRlncRNAs) for subsequent analysis (Figure 2b).

### Establishment and validation of the PRlncRNA prognostic model

**Figure 3.**
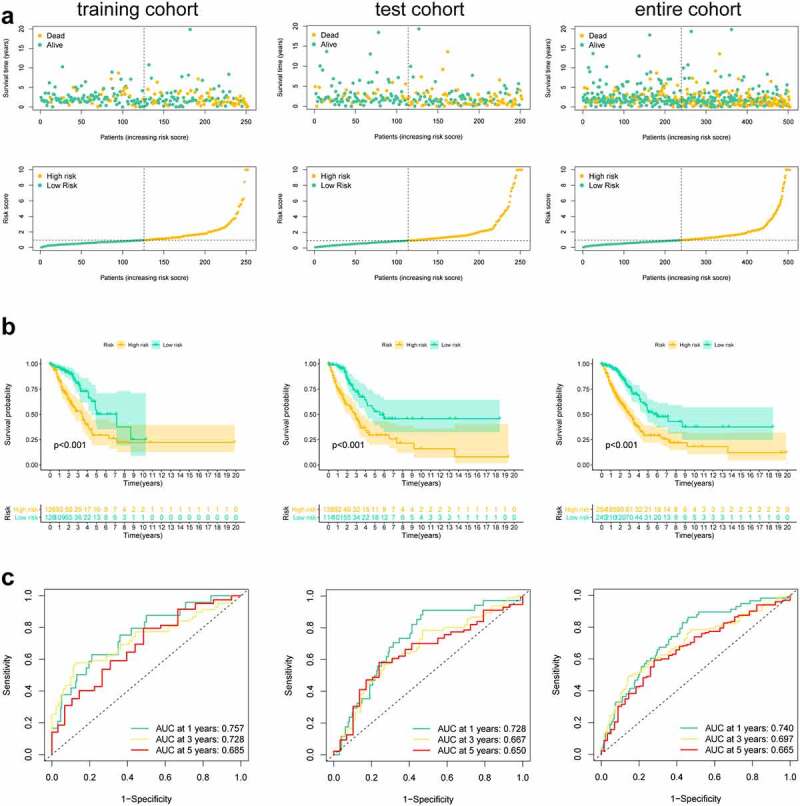
Risk score of the pyroptosis-related signature for overall survival (OS). (a) Distribution of survival and (b) risk scores of high- and low-risk patients; (c) Kaplan-Meier survival curve analysis suggests that the notable difference in OS between low-risk and high-risk score group; (d) ROC analysis for verifying model performance in the prediction of LUAD survival rates at 1, 3 and 5 years in the training cohort, validation cohort and entire cohort

First, this study classified the included cases (n = 504) into training (n = 252) and validation (n = 252) cohorts at a 1:1 ratio (Table 1). Univariate Cox regression was employed to identify 18 PRlncRNAs correlated with OS in the training cohort. Then, LASSO Cox regression was adopted to reduce the risk of overfitting using the R software ‘glmnet’ package (Figure 2c-d). Finally, multivariate Cox regression was performed to establish a prognostic pyroptosis-related risk model composed of five lncRNAs (GSEC, FAM83A-AS1, AL606489.1, AL034397.3 and AC010980.2) (Figure 2e). As shown in Supplementary Figure 1, the expression levels of five signature lncRNAs were further confirmed by paired differentiation analysis. The risk score was determined according to the following formula: Risk Score = [GSEC expression × (0.7919)] + [FAM83A-AS1 expression × (0.2422)] + [AL606489.1 expression× (0.3857)] + [AL034397.3 expression × (−0.9045)] + [AC010980.2 expression × (0.4247)]. All patients were divided into low- or high-risk groups based on the median value of the risk score. Figure 3 shows the predictive performance of our constructed five-lncRNA pyroptosis-associated prognostic model for predicting the OS of patients. The Kaplan–Meier survival curves illustrated that the OS time of high-risk LUAD patients was significantly shorter than that of low-risk patients (Figure 3b). ROC analysis was used to evaluate the predictive reliability of our prognostic signature (Figure 3c). In addition, we evaluated prognostic power in the validation cohort and the entire cohort to verify its accuracy (Figure 3c).

**Table 1.** Clinicopathologic characteristics of LUAD patients

Features	Training set	Test set	Entire set
Total	252 (100%)	252 (100%)	504(100%)
Age			
>65	136 (54.0%)	120 (47.6%)	256 (50.8%)
≤65	116 (46.0%)	132 (52.4%)	248 (49.2%)
Gender			
Male	122 (48.4%)	112 (44.4%)	234 (46.4%)
Female	130 (51.6%)	140 (55.6%)	270 (53.6%)
Stage			
I–II	204 (81.0%)	191 (75.8%)	395 (78.4%)
III–IV	48 (19.0%)	61 (24.2%)	109 (21.6%)
T Stage			
T1-T2	216 (85.7%)	221 (87.7%)	437 (86.7%)
T3-T4	35 (13.9%)	29 (11.5%)	64 (12.7%)
Unknown	1 (0.4%)	2 (0.8%)	3 (0.6%)
N			
N0	168 (66.7%)	157 (62.3%)	325 (64.5%)
N1-N3	78 (30.9%)	90 (35.7%)	168 (33.3%)
Unknown	6 (2.4%)	5 (2.0%)	11 (2.2%)
M			
M0	172 (68.2%)	165 (65.5%)	337 (66.9%)
M1	11 (4.4%)	15 (5.9%)	26 (5.1%)
Unknow	69 (27.4%)	72 (28.6%)	141 (28.0%)

### Subgroup analysis of the PRlncRNA prognostic model

**Figure 4.**
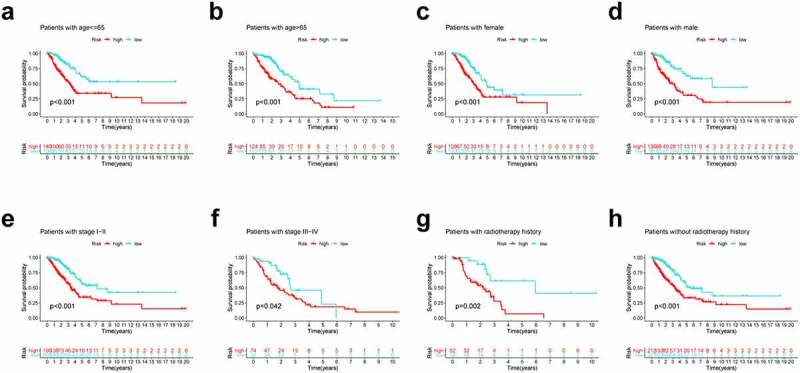
Subgroup survival analysis of OS for LUAD patients. (a) Age ≤ 65; (b) Age > 65; (c) Female; (d) Male; (e) Stage I–II; (f) Stage III–IV; (g) With radiotherapy history; (h) Without radiotherapy history

We further performed subgroup survival analysis to determine whether the prognostic model could predict OS for patients based different clinical features. These subgroups were separated by age (≤ 65 or > 65), gender (male or female), radiotherapy history and clinical stage (stage I–II or stage III–IV). As shown in Figure 4, high-risk patients exhibited inferior 5-year OS rates compared to low-risk patients according to age, gender, radiotherapy history and clinical stage.

### Construction and validation of a prognostic nomogram

**Figure 5.**
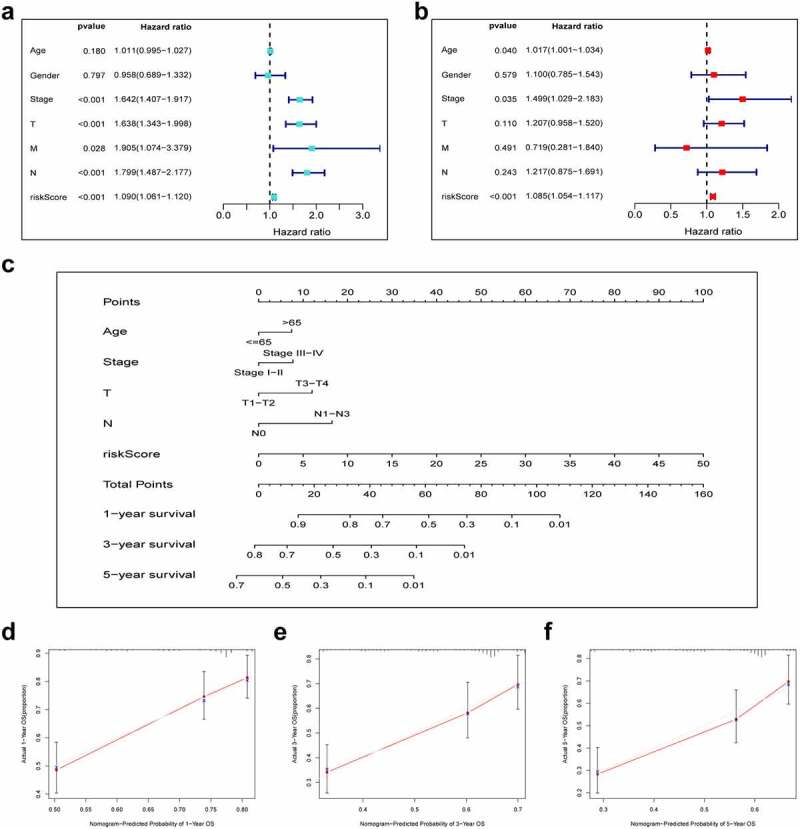
Combination of pyroptosis-related lncRNAs (PRlncRNAs) and clinical characteristics in predicting LUAD prognosis. (a) Univariate and (b) multivariate Cox regression methods for independent prognostic analysis of risk model; (c) Nomogram constructed to predict OS rates at 1, 3 and 5 years; (d-f) The nomogram calibration curves on consistency between predicted and observed 1‐, 3‐, and 5-year survival

To verify that our constructed prognostic signature could independently predict the prognosis of LUAD cases, univariate and multivariate Cox regression analyses were performed on the entire cohort. As revealed by univariate analysis, clinical stage (P < 0.001), T stage (P < 0.001), risk score (P < 0.001), N stage (P < 0.001) and M stage (P = 0.028) predicted dismal OS (Figure 5a). Moreover, our multivariate Cox regression results validated the independence of our constructed prognostic model for predicting LUAD prognosis (Figure 5b). Next, we combined the risk score and other clinicopathologic parameters to develop a novel nomogram to predict OS rates for LUAD cases at 1, 3 and 5 years, aiming to optimize the predictive accuracy of the risk model (Figure 5c). The 1-, 3- and 5-year calibration curves of our constructed nomogram fit the nomogram well for the entire cohort (Figure 5d-f).

### Functional analysis of the PRlncRNA prognostic model

**Figure 6.**
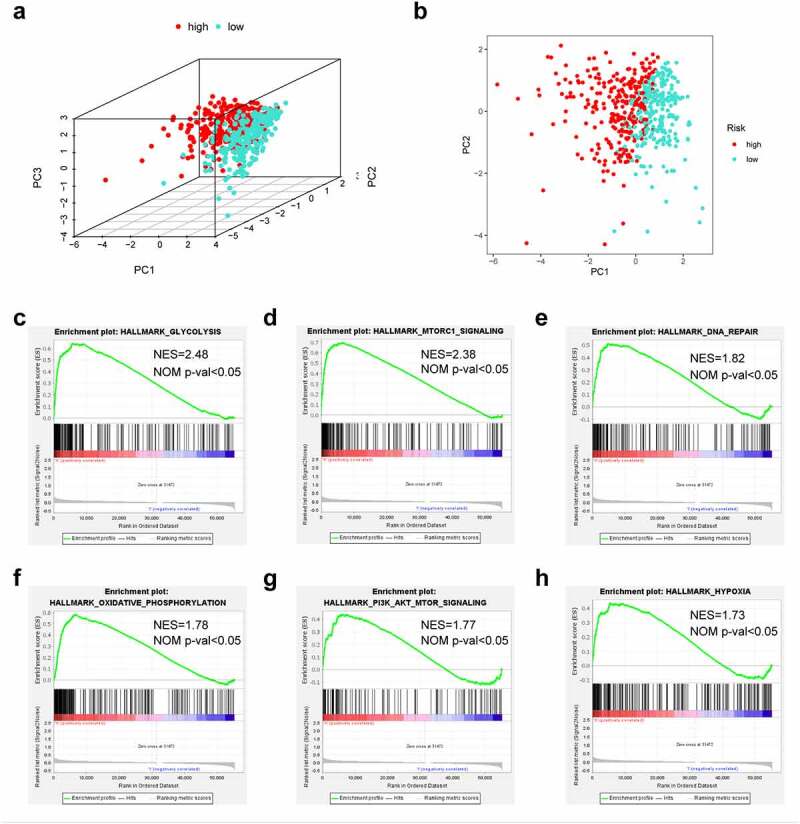
The low-risk and high-risk groups exhibited different distribution statuses and gene-set enrichment analysis (GSEA). (a) Principal components analysis (PCA) of risk groups based on the pyroptosis-associated gene sets; (b) GSEA on glycolysis; (c) GSEA on mTORC1 pathway; (d) GSEA on DNA repair; (e) GSEA on oxidative phosphorylation; (f) GSEA on PI3K/AKT/mTOR pathway; (g) GSEA on hypoxia

PCA suggested that LUAD cases of diverse groups were classified into 2 clusters (Figure 6a-b). To detect the possible biological signaling pathways associated with high-risk patients, we further applied GSEA to compare the two groups. As a result, ‘glycolysis’, ‘mTORC1 signaling’, ‘DNA repair’, ‘oxidative phosphorylation’, ‘PI3K/AKT/mTOR signaling’ and ‘hypoxia’ were significantly activated in high-risk patients (Figure 6c-h). Taken together, these findings indicate that high risk was closely correlated with processes that facilitate tumor growth and development.

### Immune infiltration analysis and mutation profile of the prognostic signature

**Figure 7.**
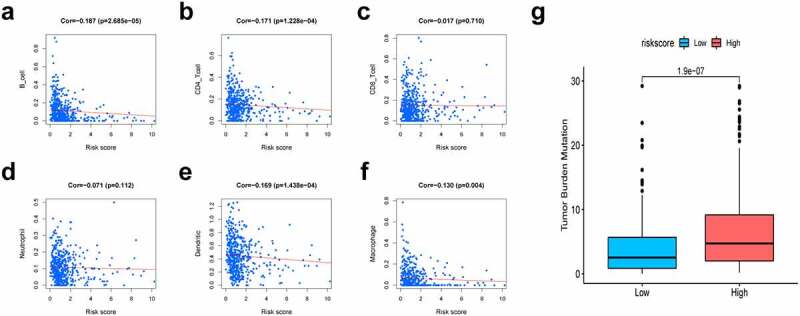
Association of risk scores with diverse immune cells and tumor mutation burden (TMB). (a) B cells (cor = −0.187); (b) CD4 + T cells (cor = −0.171); (c) CD8 + T cells (cor = −0.017); (d) Dendritic cells (cor = −0.169); (e) Macrophages (cor = −0.130); (f) Neutrophil (cor = −0.071); (g) The TMB of LUAD cases of in the high-risk and low-risk groups. Cor means the correlation value of risk scores with each immune cells

To explore the association of the constructed nomogram with the tumor immune microenvironment, this study evaluated the association of the immunocyte infiltration level with the risk score based on the TIMER database. As a result, the greater risk scores were inversely proportional to CD4 + T cells (cor = −0.171), B cells (cor = −0.187), dendritic cells (cor = −0.169, P < 0.05), and macrophages (cor = −0.130), suggesting that immune cell infiltration levels were decreased overall (Figure 7a-f). TMB has been demonstrated to be a crucial indicator for predicting the clinical benefits of immunotherapy [46]. To investigate our present model’s clinical utility for LUAD immunotherapy, the TMB of high- and low-risk patients was analyzed. We observed that high-risk cases exhibited increased TMB relative to those with low risk (Figure 7g).

### Correlation analysis between the risk group and chemotherapeutics

**Figure 8.**
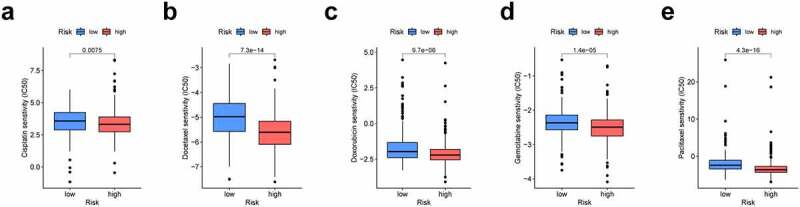
The prognosis signature used as an indicator for chemosensitivity as high-risk scores were related to half inhibitory centration (IC50) for chemotherapeutics. (a) cisplatin; (b) docetaxel; (c) doxorubicin; (d) gemcitabine; (e) paclitaxel

Chemotherapy still plays a vital role in treating LUAD patients. Based on the LUAD dataset of TCGA, we investigated the associations between risk group and the efficacy of common chemotherapeutics in treating patients. The findings indicated that the high-risk group exhibited a lower IC50 for cisplatin, docetaxel, doxorubicin, gemcitabine and paclitaxel (P < 0.05), suggesting that our proposed risk signature can be used as a potential indicator of drug sensitivity (Figure 8).

### Knockdown of GSEC attenuates LUAD cell proliferation and promotes pyroptosis

**Figure 9.**
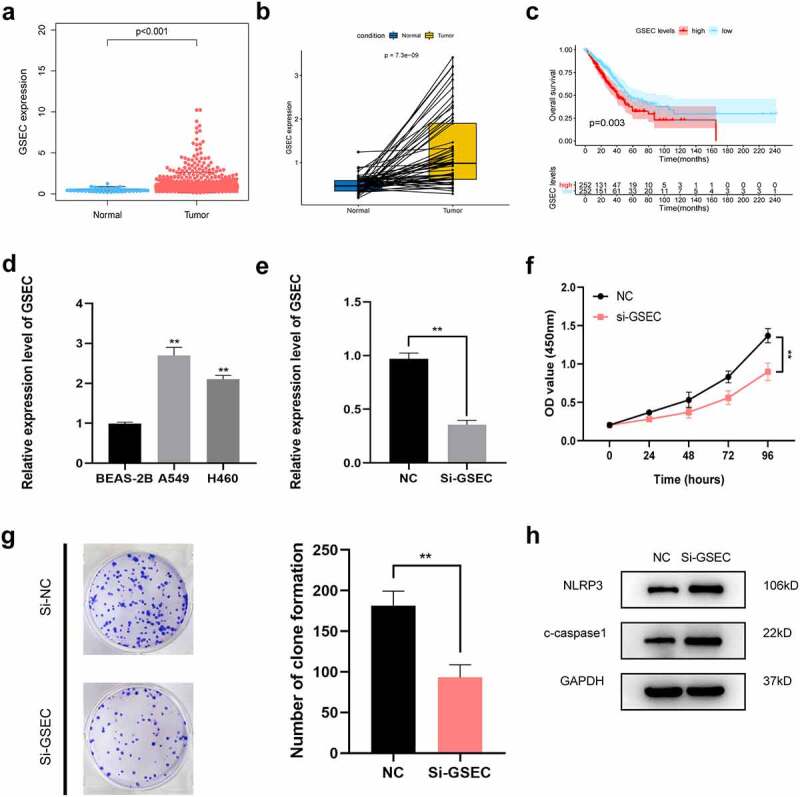
Effects of inhibiting the expression of GSEC on LUAD cell proliferation and pyroptosis. (a) Differential expression of GSEC between cancer and non-carcinoma samples; (b) Paired differentiation analysis on GSEC expression between cancer and non-carcinoma samples collected in one patient; (c) Kaplan-Meier survival analysis for LUAD patients with different GSEC expression; (d) GSEC expression increased within H460 and A549 cells relative to BEAS-2B; (e) GSEC was downregulated in A549 using siRNAs; (f-g) The proliferation of A549 cells transfected with siRNA against GSEC were measured using CCK8 assays and colony formation assays; (h) Western Blot was carried out to examine NLRP3 and cleaved caspase-1 (*p < 0.05; **p < 0.01; ***p < 0.001)

We chose GSEC to confirm our signature. First, the expression profile and prognostic value of GSEC were evaluated using the TCGA dataset. As a result, GSEC expression was remarkably increased in LUAD samples compared to non-carcinoma samples (Figure 9a-b). According to Kaplan–Meier analysis results, increased GSEC levels predicted poor OS in LUAD (Figure 9c). Next, PCR assays demonstrated that GSECs were highly expressed in LUAD cell lines (A549 and H460) compared to BEAS-2B cells (Figure 9d). Next, we used A549 cells to explore the functional role of GSEC. As shown in Figure 9e, GSEC expression was markedly decreased in A549 cells in response to si-GSEC transfection. CCK-8 assays demonstrated that si-GSEC-transfected A549 cells exhibited a markedly decreased growth rate relative to the negative control (Figure 9f). Moreover, colony formation assays also demonstrated that silencing GSEC significantly suppressed the proliferation of A549 cells (Figure 9g). Western blot analysis illustrated that downregulation of GSEC increased NLRP3 and cleaved caspase-1, indicating the potential role of GSEC on LUAD cell pyroptosis (Figure 9h).

## Discussion

Lung cancer ranks second among health problems and is the major cause of cancer-associated mortality worldwide. LUAD is considered to be the most common subtype in nonsmokers. The prevalence of LUAD is rapidly increasing with the development of anti-smoking movements. Although headway has been made in cancer treatment, the overall survival of LUAD remains disappointing due to a lack of reliable early prognostic indicators. Pyroptosis, a novel accepted form of non-apoptotic cell death, was proved to be a double-edged sword for tumor progression and cancer therapy. Under the stimulation of a great deal of inflammatory cytokines released by pyroptotic cell, normal cells could be transformed into cancer cells [36]. Alternatively, the development of tumor pyroptotic death could make pyroptosis a treatment target for cancer [19]. Numerous studies suggest that lncRNAs play central part in the sustainment of various biological activities in tumor, such as cell pyroptosis. As discovered by Tan et al., HOTTIP, a crucial oncogenic driver in various cancers, could block pyroptosis by binding with miR-148a-3p and further positively enhancing AKT2 [47]. In liver cancer, NLRP3-related pyroptosis pathway suppressed by SNHG7 through regulation of miR-34a/SIRT1 ceRNA axis [48]. In addition, MEG3 was found to activate cisplatin-induced cellular pyroptosis by promoting NLRP3/caspase-1/GSDMD axis, implying that MEG3 could be effective therapeutic target of breast cancer [49]. Therefore, it is essential to identify robust pyroptosis-related signatures to enhance the prognostic prediction of LUAD patients. This work successfully established a prognostic risk signature based on PRlncRNAs for predicting the overall survival in patients with LUAD. Furthermore, we initially investigated the oncogenic role of GSEC in LUAD and found that suppression of GSEC may inhibit proliferation and facilitate pyroptosis in LUAD cells.

In this study, the Pearson correlation method and differential expression analysis were used to identify 84 DEPRlncRNAs. Next, these lncRNAs were selected to develop a five-PRlncRNA signature based on the training set. Then, we adopted ROC analysis to evaluate the predictive performance of our constructed risk model. The AUCs of the ROC curves for 1-, 3-, and 5-year OS in our proposed model were 0.757, 0.728 and 0.848, respectively. Furthermore, we utilized ROC curves to compare the predictive ability of our present risk signature to other signatures. We observed that our risk signature attained consistently outstanding predictive power compared to other published pyroptosis‑based prognostic models in LUAD [50]. Moreover, we confirmed that our signature exhibited strong independent prognostic ability for OS. Finally, we constructed a nomogram that integrated the risk score and clinical characteristics to enhance the prediction of LUAD prognosis.

Our present pyroptosis-related signature consists of five PRlncRNAs, which were remarkably correlated with OS in LUAD patients. Among these five lncRNAs, GSEC, FAM83A-AS1, AL606489.1 and AC010980.2 are potentially dangerous lncRNAs, but AL034397.3 is a potentially protective lncRNA. In this study, a number of lncRNAs in the risk model, such GSEC, FAM83A-AS1, AC010980.2 and AL034397.3, were suggested to exert vital roles in regulating different cancers, while AL606489.1 was identified for the first time. GSEC was shown to participate in cancer growth and development. In osteosarcoma, GSEC boosts proliferation and metastasis through the miR-588/EIF5A2 axis with sponge activity [51]. As suggested by Matsumura et al., GSEC was highly expressed within colorectal cancer (CRC) samples and regulated tumor migration by targeting DHX36 [52]. FAM83A-AS1 exerts a carcinogenic effect on LUAD, esophageal cancer and hepatocellular carcinoma. As discovered by Xiao et al., FAM83A-AS1 promotes the development of LUAD by regulating MMP14 expression and binding to miR-150-5p [53]. In addition, FAM83A-AS1 may also strengthen the pre-mRNA stability of FAM83A to improve the metastatic ability of lung adenocarcinoma [54]. In esophageal cell squamous carcinoma, FAM83A-AS1 downregulation regulates the miR-214/CDC25B axis to suppress tumor cell growth, invasion and migration [55]. Furthermore, He and colleagues reported that FAM83A-AS1 was expressed in HCC cells and tissues, suggesting that increased FAM83A-AS1 expression enhances tumor proliferation and migration and represses apoptosis by interacting with NOP58 [56]. Our results are in line with these studies, suggesting that GSEC and FAM83A-AS1 are risk factors (HR > 1) in LUAD. Interestingly, AC010980.2 was identified to be closely associated with immunity, autophagy and ferroptosis in LUAD and may represent an oncogene in a risk model for predicting the prognosis of patients with LUAD [57–59]. In addition, Jin et al. used AL034397.3 to establish an immune-related risk model that improved the prediction of LUAD prognosis [60].

To further detect the underlying functional mechanisms of the signature, we performed GSEA. The high-risk group exhibited significantly activated ‘glycolysis’, ‘mTORC1 signaling’, ‘DNA repair’, ‘oxidative phosphorylation’, ‘PI3K/Akt/mTOR signaling’, and ‘hypoxia’ compared to the low-risk group. Aerobic glycolysis is recognized as the characteristic metabolic pathway of cancer. Efficient glucose utilization by malignant tumors is associated with high proliferation, aggressiveness, and self-renewal capacity [61]. Numerous studies have demonstrated a link between DNA damage repair and glycolysis, which are interdependent and promote the uncontrolled proliferation and survival of tumor cells [62]. Thomas M Ashton et al. revealed that oxidative phosphorylation is commonly upregulated in tumor cells with high metastatic and tumorigenic potential, including LUAD [63]. In a hypoxic microenvironment, activated hypoxic-inducible factor-1α may promote epithelial to mesenchymal transformation (EMT), thereby increasing invasion, tumor stem cell-like phenotypes, and chemoradiotherapy resistance [64]. MTORC1 is the downstream effector in oncogenic pathways with frequent mutations, such as the MAPK pathway (excessively activated in diverse human cancers) and the PI3K/Akt pathway [65]. Upregulation of the mTOR pathway is observed in up to 90% of lung adenocarcinoma patients [66]. Immune cell infiltration has an important effect on LUAD survival. The results of immunocyte infiltration analysis revealed that the risk score exhibited a negative correlation with the infiltration of B cells, CD4 + T cells, DCs and macrophages. It has been previously shown that increased CD4 + T cells are a favorable independent prognostic factor for NSCLC [67]. B cells may restrain tumor cells and reduce the incidence of occult micrometastases, resulting in prolonged survival by limiting further tumor spread [67]. Romain Remark et al. summarized several works regarding the association of immune cells with NSCLC survival, suggesting that B-cell density is an indicator of better prognosis [68]. With the increase in risk score, infiltration levels of B cells and CD4 + T cells decreased, consistent with the reduced survival times of high-risk patients.

To better assess the clinical feasibility of the risk model, we analyzed the efficacy of the presented model in immunotherapy according to the tumor mutation burden (TMB). Based on these findings, high-risk LUAD patients exhibit higher TMBs than low-risk patients, suggesting that our signature could be a potential index for evaluating the efficacy of immunotherapy in patients with LUAD. In addition to immunotherapy, we also identified the relationship between the signature and chemotherapy sensitivity of patients. Collectively, these discoveries may offer prospective treatment alternatives for LUAD patients.

Moreover, the functional phenotypic role of GSEC was investigated by experimental studies. We verified expression levels of GSEC between human lung epithelial cells and human lung adenocarcinoma cell lines. *In vitro* analysis showed that inhibition of GSEC blocked proliferation and activated pyroptosis in A549 cells by triggering the NLRP3 inflammasome and cleaved caspase-1, indicating that GSEC might be a potential pyroptosis-related lncRNA in LUAD.

In our analysis, we found that the integrated pyroptosis-related signature and clinical factors heightened the predictive reliability of prognosis relative to the TNM staging system, which may undergo routine application in the future. In addition, the proposed signature offers improved clinical utility for immunotherapy strategies and chemotherapy drug selection in LUAD patients.

## Limitations

There are some shortcomings in our present study. First, the original dataset for setting up the lncRNA-related model was merely retrieved from the TCGA database. Our risk model needs to be validated for reliability and accuracy in other external datasets and large-scale clinical cohorts. Second, the mechanism by which pyroptosis regulates the precise process of LUAD is still not known and needs to be elucidated through additional studies.

## Conclusions

The present study first developed a five-PRlncRNA signature that offers valuable clinical application for accurate prognostic forecasting. Our signature provides insights into personalized treatment for LUAD patients.

## Supplementary Material

Supplemental Material

## Data Availability

Publicly available datasets were analyzed in this study. These data can be found here: TCGA (https://portal.gdc.cancer.gov/).

## Highlights

- A novel pyroptosis-related lncRNA signature was established.
- Our signature improves the prediction of lung adenocarcinoma prognosis.
- Pyroptosis might be associated with the immune response in lung adenocarcinoma.
