# Supplementary material for: A novel pyroptosis-related lncRNA signature for prognostic prediction in patients with lung adenocarcinoma
Source: Bioengineered. 2021 Sep 7;12(1):5932–49. doi: 10.1080/21655979.2021.1972078 (PMC8806662; doi:10.1080/21655979.2021.1972078)
Supplement: Supplemental Material [file KBIE_A_1972078_SM2805.zip › supplementary/Supplementary Figure 1.docx]

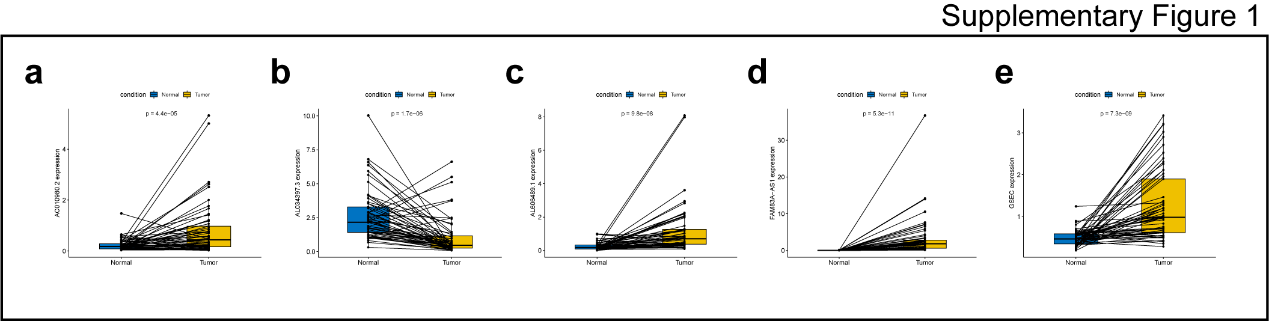


**Supplementary Figure 1** paired differentiation analysis for expression levels of five signature lncRNAs in the normal and LUAD tumor sample deriving from the same one patient. (a) AC010980.2. (b) AL034397.3. (c) AL606489.1. (d) FAM83A-AS1. (e) GSEC.
