# Supplementary figures and images for: A novel pyroptosis-related lncRNA signature for prognostic prediction in patients with lung adenocarcinoma
Source: Bioengineered. 2021 Sep 7;12(1):5932–49. doi: 10.1080/21655979.2021.1972078 (PMC8806662; doi:10.1080/21655979.2021.1972078)

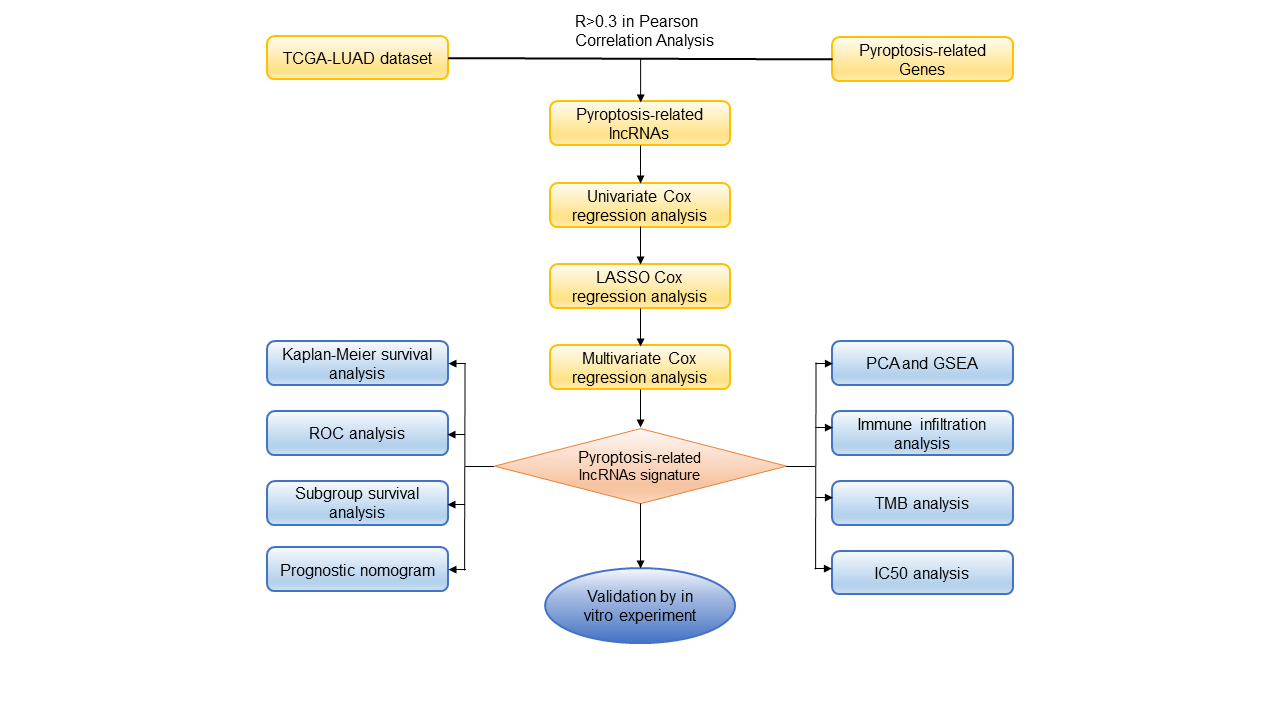

Supplement: Supplemental Material [file KBIE_A_1972078_SM2805.zip › supplementary/flow chart.tif]
